# LGI1-antibody encephalitis is characterised by frequent, multifocal clinical and subclinical seizures

**DOI:** 10.1016/j.seizure.2017.05.017

**Published:** 2017-08

**Authors:** Sidra Aurangzeb, Mkael Symmonds, Ravi K. Knight, Robin Kennett, Tim Wehner, Sarosh R. Irani

**Affiliations:** a Department of Clinical Neurophysiology, Oxford University Hospitals, John Radcliffe Hospital, Oxford, OX3 9DU, United Kingdom; b Autoimmune Neurology Group, Nuffield Department of Clinical Neurosciences, University of Oxford, John Radcliffe Hospital, Oxford, OX3 9DU, United Kingdom; c Department of Clinical Neurophysiology and Clinical, National Hospital for Neurology and Neurosurgery, 23 Queen Square, London, WC1N 3BG, United Kingdom

**Keywords:** Autoimmune encephalitis, Leucine-rich glioma inactivated-1, Autoantibody, Neuroimmunology, Epilepsy

## Abstract

**Purpose:**

To describe clinical and electrographic characteristics of seizures LGI1-antibody encephalitis, and their correlations with two-year outcomes.

**Methods:**

Video-electroencephalography recordings were performed on a cohort of 16 consecutive patients with LGI1-antibodies from two UK neuroscience-centers over five-years.

**Results:**

From 14 of 16 patients (13 males; age-range 53–92 years), 86 faciobrachial dystonic seizures were recorded at a median frequency of 0.4 per hour (range 0.1–9.8), and ictal EEG changes accompanied 5/86 events. In addition, 11/16 patients showed 53 other seizures – subclinical (n = 18), motor (n = 16), or sensory (n = 19) – at a median of 0.1 per hour (range 0.1–2) associated with temporal and frontal discharges. The sensory events were most commonly thermal sensations or body-shuddering, and the motor events were frequently automatisms or vocalisations. Furthermore, multifocal interictal epileptiform discharges, from temporal, frontal and parietal regions, and interictal slow-wave activity were observed in 25% and 69% of patients, respectively. Higher observed seizure frequency correlated with poorer functional recovery at two-years (p = 0.001).

**Conclusions:**

Multiple frequent seizure semiologies, in addition to numerous subclinical seizures and interictal epileptiform discharges, are hallmarks of LGI1-antibody encephalitis. High overall seizure frequency may predict more limited long-term recovery. These observations should encourage closer monitoring and proactive treatment of seizure activity in these patients.

## 1 Introduction

LGI1-antibodies are closely associated with a limbic encephalitis (LE) which is characterised by confusion, disorientation and seizures, frequently with medial temporal lobe inflammation on imaging [1]. The seizures include typical medial temporal lobe events [2], [3], [4], and more distinctive semiologies including bradycardia, piloerection, and faciobrachial dystonic seizures (FBDS) [2], [4], [5], [6], [7]. These multiple seizure descriptions appear in several separate reports, largely based on retrospective histories. As patients often show some amnesia for the acute illness, and these reports lack the gold-standard of video-EEG monitoring [2], [5], [8], our aim was to systematically describe and quantify the electroclinical characteristics of seizures in patients with LGI1-antibodies, with a focus on seizure localisation, semiology and frequency, from consecutive patients attending video-EEGs.

## 2 Materials and methods

Sixteen consecutive adults with LGI1-antibody encephalitis and clinco-electrographic events during video-EEG recordings were seen at the author’s two institutions between 2007 and 2012. Ethical approval for patient consent and data collection was available (references 07/Q1604/28, 16/YH/0013 and OUH10563). Forty-one EEGs from these 16 patients were reviewed by two consultant clinical neurophysiologists (TW and RK), and findings were systematically recorded in a database formatted as Table 1.

**Table 1:** Clinical and electrographic characteristics of patients with LGI1-antibodies. LGI1-antibody level (end-point dilution shown; * = additional CASPR2-antibody positivity), A = arm, F = face, L = leg involvement during FBDS; ^a^ = lateralisation (R right; L left; BL bilateral); ^b^ = distribution (CT centrotemporal; Temp temporal; FT Frontotemporal; FC Frontocentral; FCP Fronto-Centro-Parietal)**;**^c^ = two patients had sensory aura preceding some motor events; ^p^ = patients who underwent prolonged recordings.

			FBDS			Motor/ subclinical seizures		Sensory seizures		Frequency (motor & sensory seizures)
Age (years)	Sex	LGI1- antibody level	Number per hour	Arm, face and/or leg involved	Ictal EEG changes	Semiology^a^	Ictal EEG^a,b^	Semiology	Ictal EEG^a,b^	Number per hour
71	M	540	Not captured		N	Clonic L arm and neck (3/4), automatisms (1/4)	L CT (n = 4)	–	–	1.4
67	M	14580	5.4	AF,AFL and L	N	–	–	Fear, tingling cold sensation, and flushing (n = 1)	Nil	0.6
61^p^	M	4860	4.2	A	N	–	–	–	–	
78^p^	M	4860	0.4	AF	N	Posturing,lip smacking, blinking and tachycardia (3/3)	R Temp (n = 3)			0.1
92^p^	M	14580	0.1	AFL	Y	–	–	–	–	–
63	M	1620	Not captured		N	–	–	Warm surge (n = 1)	Nil	2
69^p^	M	14580	0.2	AF	N	–	–	–	–	–
56^c,p^	M	14580	0.1	AF	N	L shoulder twitching, throat clearing, lip smacking, L hand posturing (1/7)	L FT (n = 1); FC (R 2/3, L 1/3), R Temp (n = 3)	Tingling (n = 2)	BL FC (1/2)	0.1
68	M	1620	2.4	AF and F	N	–	–	–	–	–
76^p^	M	1620	0.2	AFL, and F	N			Pain R face (n = 1)	Nil	0.1
64	F	4860	4.2	AFL, AF and L	Y	Arousal (1/6), no clinical change in 5/6	R FT (n = 6)	–	–	1.5
63	F	4860*	9.8	AFL	Y	Oral automatisms (1/1)	R Temp (n = 1)	–	–	0.1
69^c,p^	M	4860*	0.2	AFL, AF and LL	N	Nocturnal arousals (3/6), no clinical change (2/6); L hand twitching (n = 1/6)	L FT (n = 5)	Cold sensation (n = 9); lip quivering (n = 3);)	L Temp (1/9) and L FCP (1/9)	0.7
66^p^	M	4860*	0.4	AFL	N	–	–	–	–	0.1
64	F	14580	5.4	AF	N	Vocalisations, unresponsive, automatisms, post ictal confusion (8/8); L Head version (n = 3/8)	L Temp (n = 6) and R FT (n = 2)	Body shuddering and goosebumps (n = 2)	Nil	0.1
53	M	4860	0.1	AF	N	–	–	Body shuddering (n = 3)	Nil	0.1

## 3 Results

### 3.1 Clinical and EEG characteristics

As shown in Table 1, 13/16 (81%) patients were male, and median age was 67 years (53–92). At times of EEG recordings, 15/16 (94%) patients had cognitive impairment, and were receiving AEDs (n = 16) and immunotherapies (n = 14). Eight of 16 patients underwent prolonged video telemetry (24–120 h); eight had EEGs of 20–30 min duration with video recordings.

### 3.2 Clinical and EEG features of FBDS

From 14 of 16 patients, 86 FBDS were recorded (median 6 per patient, range 1–28, Table 1). The face and arm were both involved in 70 attacks, of which 12 also involved the leg. A further four events involved the face alone, six the arm alone, and six events exclusively involved the leg. Twenty-two of 86 FBDS showed associated ictal features including dysphasia, fear, oral automatisms, vocalisations or loss of awareness. FBDS occurred during wakefulness (n = 48), from sleep (n = 32), and from drowsiness (n = 6), and their frequency varied from 0.1–9.8 per hour (median = 0.4).

EEG showed prominent muscle artefact (lasting 0.5 to 1.6 s) during 81/86 recorded FBDS. In the remaining five events (6%), three recorded from the same patient, preceding rhythmic delta wave activity was observed at onset over the left frontotemporal region (Supplementary Fig. 1). These were followed by muscle artefact and generalised EEG attenuation, and around seven seconds later, sharply contoured slow wave activity appeared and persisted for 30 s. In the other two events, preceding slow wave activity was seen in the left frontocentral electrodes before muscle artefact, and the FBDS were around five seconds in duration with prolonged post-ictal confusion (Video 1).

### 3.3 Motor, sensory and subclinical seizures other than FBDS

A variety of semiologies other than FBDS were also captured at overall frequencies similar to the FBDS (median = 0.1, range 0.1 to 2 per hour, Table 1). In total, 53 seizures other than FBDS were captured in 12 patients: 18/53 were subclinical, 16/53 had motor features and 19/53 showed sensory semiologies. Overall, accompanying electrographic changes were present in 37 of 53 events (70%).

The 18 subclinical seizures (example in Supplementary Fig. 2) showed ictal evolution in the frontotemporal (11/18, 61%), temporal (3/18, 16%) and frontocentral regions (4/18, 22%), within either the right (62%) or left (38%) hemispheres.

The motor semiologies (example in Video 2) showed features including automatisms (13/16, 81%), vocalisations (8/16, 50%), clonus (4/16, 25%), dystonic posturing (4/16, 25%), version (3/16, 18%) and eye blinking (3/16, 18%). All motor events were accompanied by ictal EEG changes seen in the temporal (62%), frontotemporal (18%), centrotemporal (18%) and frontocentral (18%) regions, within the left (62%) or right (38%) hemispheres.

The 19 sensory events were described as thermal alterations (n = 11, 58%, Video 3), body-shuddering (n = 5, 26%), tingling (n = 3, 16%), lip quivering (n = 3, 16%), goosebumps (n=2, 11%) pain (n = 1) and flushing (n = 1), and associated with less frequent EEG changes involving bilateral frontocentral (n = 1), left frontocentroparietal (n = 1, Supplementary Fig. 3) and left temporal (n = 1) regions in 3 of 19 events. Autonomic and emotional features, typically tachycardia (n = 3), flushing (n = 1) and fear (n = 1) were associated with both motor and sensory seizures.

### 3.4 Interictal EEG changes

**Fig. 1:**
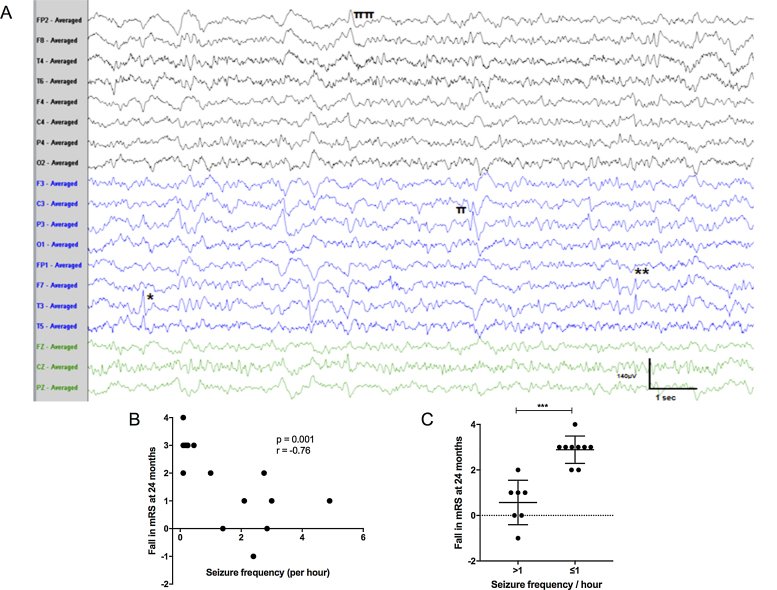
Seizures in LGI1-antibody encephalitis. A. EEG showing multifocal epileptiform discharges independently over the left mid to posterior temporal (*), left frontotemporal (**), left centroparietal (π) and right hemispheric (ππ) electrodes, on the background of excess of slow wave activity (sensitivity 7microV/mm, time base 30 mm/s). B. Relationship between 24-month fall in modified Rankin Score and observed seizure frequency (Spearman’s correlation r = −0.76, p = 0.001). C. Seizure frequency over one per hour associates with a more limited reduction in modified Rankin Score (mRS) at two-year follow-up (Mann Whitney *U* test p = 0.0025).

Interictal epileptiform discharges were seen in nine EEGs from 4 of 16 (25%) patients (Fig. 1A). The discharges were typically multifocal, and most prominent over the temporal regions (n = 7/9; right = 1, left = 3, bilateral = 3; 77%), followed by centro-temporo-parietal region in 2/9 (22%) and right frontocentral, midline central and right frontotemporal electrodes in one patient each (11%). In addition, two patients developed asymptomatic repetitive sharp waves over the whole right hemisphere, lasting up to 13 s, as a brief periodic lateralised discharge, but without acceleration or deceleration suggestive of seizure activity (Supplementary Fig. 4).

Interictal EEGs in 5/16 patients (31%) were normal, despite cognitive impairment in these five patients. Overall, mean background frequency was 8.5 ± 2.2 Hz (range 3–12). In the remaining 11/16 patients, interictal EEG abnormalities were observed in all their 24 EEGs. Excess slow wave activity was mild (background EEG 6–7 Hz without delta activity; n = 9, 37%), moderate (4–7 Hz with infrequent delta wave activity; n = 7, 29%), and severe (delta wave activity throughout recording; n = 8, 33%). The slowing was diffuse in 14/24 (58%) and focal slow wave activity was seen in 10/24 (42%), over temporal regions (n = 10, left, right and bilaterally) plus bifrontotemporal and bifrontal regions, in two patients each.

### 3.5 Long-term clinical correlations

The cumulative frequency of all observed seizures at time of video-EEG showed a negative correlation with the overall functional improvement (fall in modified Rankin Score) at two-year follow-up (Fig. 1B; Spearman’s r = −0.76, p = 0.001), and more than one seizure per hour at time of video-EEG accounted for all patients with limited recoveries (Fig. 1C, Mann Whitney *U* test, p = 0.0025). No similar association was seen with age, sex, or time to medication (data not shown).

## 4 Discussion

Patients with LGI1-antibody encephalitis have a striking number of frequent, multifocal seizure localisations with multiple semiologies, in addition to faciobrachial dystonic seizures and numerous subclinical seizures. Overall, the clinically-apparent seizures were observed at a median of around 12 per day, despite AEDs and immunotherapies. Sensory semiologies were as common as motor events, and were most frequently thermal or shivering sensations. By contrast to the motor seizures, the sensory seizures were infrequently associated with EEG changes. The observed temporal, frontal and parietal electrical activities extend the pathology of LGI1-antibody encephalitis beyond the medial temporal lobes. Overall, a high seizure burden in the acute phase related to poorer long-term recovery: patients observed to have more than one seizure per hour showed limited improvements in function at two years.

Dyscognitive, autonomic, motor, gelastic and fearful seizures have previously been noted in LGI1-antibody encephalitis patients across studies with varying methodologies and inclusion criteria [2], [8]. Also, other studies of LGI1-antibody positive patients have noted subclinical seizures [2], [9], [10]. In one of these reports, the seizures were often triggered by hyperventilation [10], with few interictal epileptiform discharges. By contrast, we found interictal epileptiform discharges in a significant number of patients, and perhaps this variation is explained by the different timings of EEGs within the disease course. Furthermore, our study used direct video observations to report the clinical and EEG findings in a cohort with LGI1-antibodies, which has objective advantages over patient- or relative-reporting in previous studies. However, as video-EEGs were often performed once behavioural disturbances had largely settled after treatment, our study likely under-emphasised the maximal frequency of seizures documented in other studies [2], [5], [8].

Taken together with available reports, our findings suggest the unifying possibility that a combination of multiple motor semiologies, prominent thermal sensations, ictal piloerection, ictal cardiac arrhythmias, and frequent subclinical seizures should alert the clinician to the possibility of underlying LGI1-antibodies. Identification of such patients is, of course, often also aided by the highly-distinctive semiology of FBDS [2], [9], [10]. Clinical recognition of this variety of semiologies alone should prompt rapid consideration of immunotherapies, especially if they evolve over a short duration (e.g.< 3 months) [11].

The observed clinical and electrical multifocality suggests a diffuse electrical hyperexcitability in patients with LGI1-antibodies, extending the disease pathophysiology to distributed cortical regions beyond the medial temporal lobes and motor cortex [2]. In addition, the rare ictal EEG changes with FBDS strongly implicate basal ganglia circuits in this dystonic syndrome [2], [5], [9], [12]. Both observations are consistent with the widespread distribution of LGI1 in the brain. The high frequency of clinical and subclinical seizures suggests routine consideration of prolonged EEG monitoring to guide more aggressive seizure suppression in LGI1-antibody encephalitis. This is especially important given the observed interaction between frequent seizures and long-term outcomes. Indeed, the high and widespread seizure burden in this condition may relate to the development of the limbic encephalopathy, and is consistent with the hypothesis that early termination of seizures may prevent future cognitive impairment in these patients [12]. This theory should motivate clinicians to actively recognise the described semiologies and EEG appearances, and encourage early treatment.

## Author contributions

All those designated as authors meet all 4 criteria for authorship according to the guidelines of the International Committee of Medical Journal Editors (ICMJE) and all coauthors have reviewed and approved the contents of the manuscript.

## Funding sources

SRI is supported by a Wellcome Trust Intermediate Fellowship, BMA Research Grants – Vera Down grant, the Fulbright UK-US commission and the MS society. Research in the Neuroimmunology Lab is supported by the Oxford NIHR Biomedical Research Centre.

## Conflict of interest

SRI is a coapplicant and receive royalties on patent application WO/2010/046716 entitled ‘Neurological Autoimmune Disorders'. The patent has been licensed to Euroimmun AG for the development of assays for LGI1 and other VGKC-complex antibodies.
