# Supplementary material for: LGI1-antibody encephalitis is characterised by frequent, multifocal clinical and subclinical seizures
Source: Seizure. 2017 Aug;50:14–7. doi: 10.1016/j.seizure.2017.05.017 (PMC5558811; doi:10.1016/j.seizure.2017.05.017)
Supplement: Supplementary file 1 [file mmc1.docx]

**Supplementary Figure 1. Faciobrachial dystonic seizure with additional ictal features.** A. Delta wave activity was noted over the left frontotemporal region at onset (*). Subsequently, a muscle jerk (**) is followed by generalised EEG attenuation and then rhythmical, irregular, slow wave activity with sharp components in the left frontotemporal area. This persisted for about 30 seconds. Onset of faciobrachial dystonic seizure indicated by downward arrow. This patient also had confusion, oral automatisms and expressive dysphasia after the faciobrachial dystonic seizure (Video 1). Sensitivity 10microV/mm, time base 15 mm/sec.

**Supplementary Figure 2. Subclinical seizure.** Subclinical seizure with ictal onset from sleep beginning as incremental irregular, mixed frequency fast alpha/beta activity over the left centrotemporal region. This persisted for 90 seconds. Sensitivity 7microV/mm, time base 15 mm/sec.

**Supplementary Figure 3. Ictal activity during sensory symptoms**. Patient (Video 3) wakes up with sudden onset cold sensation and lip quivering (*) and EEG showed lateralised rhythmical delta activity (LRDA) over the left frontocentroparietal electrodes. Sensitivity 7microV/mm, time base 30 mm/sec.

**Supplementary Figure 4.** Brief periodiclateralised discharges with a pseudo-periodic lateralized discharge (PLD)-like pattern over the right frontocentroparietal region without clinical manifestations. Sensitivity 7microV/mm, time base 15 mm/sec.

**Video 1**. Faciobrachial dystonic seizure (FBDS). FBDS followed immediately by development of oral automatisms and speech difficulty (corresponding to Supplementary Figure 1).

**Video 2.** Focal seizure with motor semiology. Seizure begins with manual automatisms, followed by clonic left hand/arm jerking, which quickly spreads to include the neck and continues for about one minute.

**Video 3.** Sensory seizure where patient reports a “cold-rush” from his knee to neck associated with subtle lip quivering (corresponding to Supplementary Figure 3)
