# Supplementary figures and images for: LGI1-antibody encephalitis is characterised by frequent, multifocal clinical and subclinical seizures
Source: Seizure. 2017 Aug;50:14–7. doi: 10.1016/j.seizure.2017.05.017 (PMC5558811; doi:10.1016/j.seizure.2017.05.017)

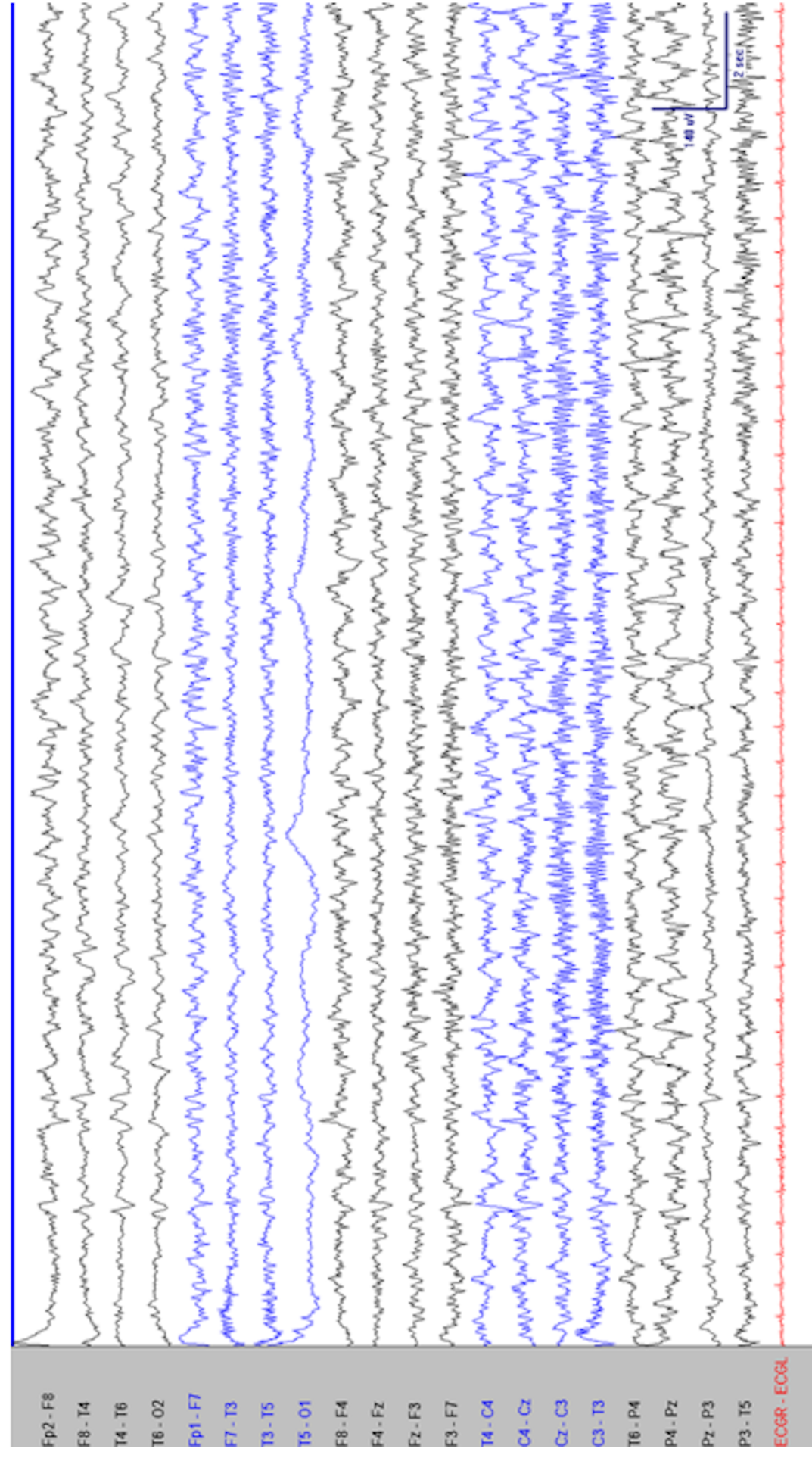

Supplementary Figure 2

Supplement: Supplementary file 2 [file mmc2.pdf]

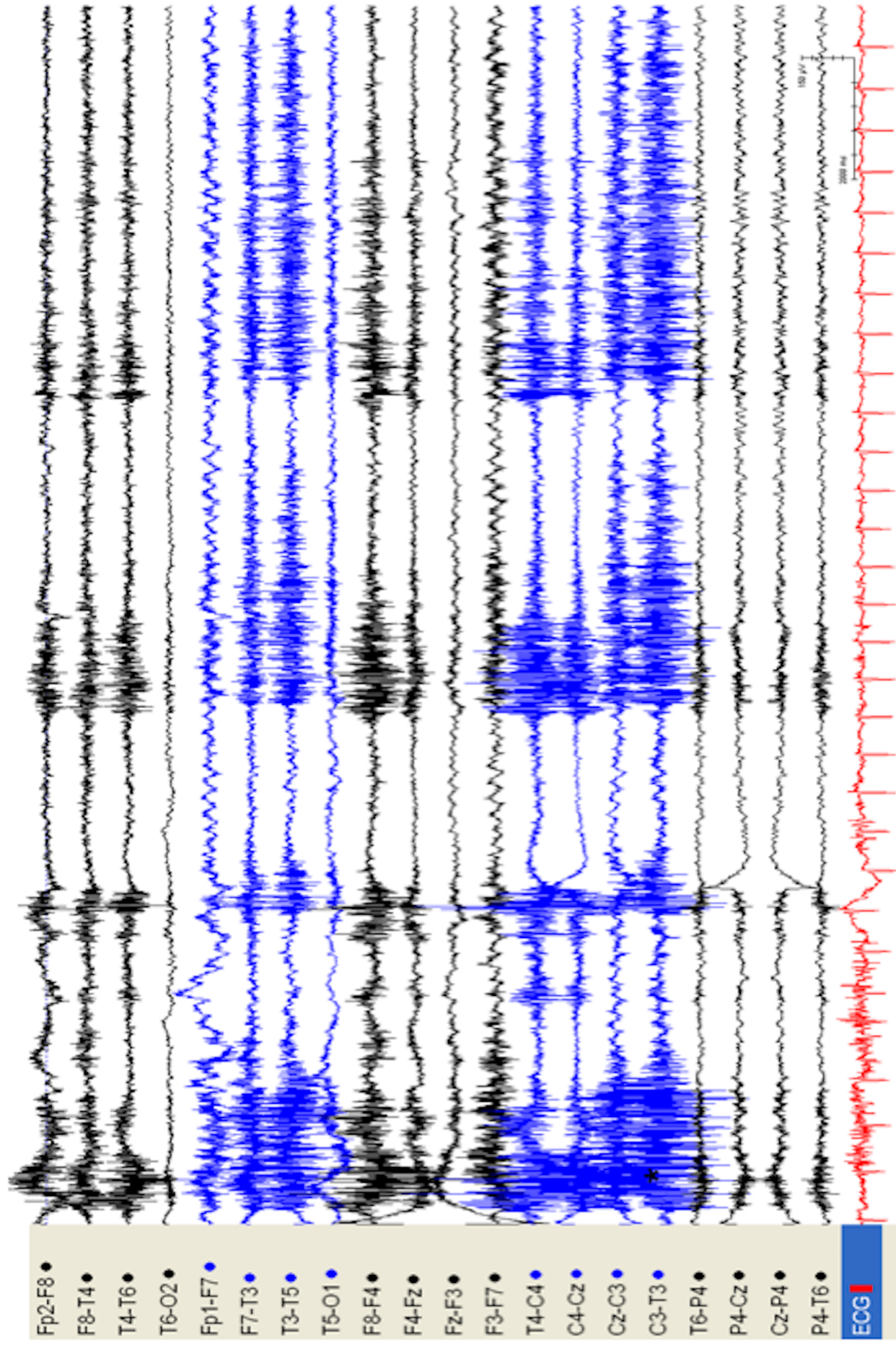

Supplementary Figure 3

Supplement: Supplementary file 3 [file mmc3.pdf]

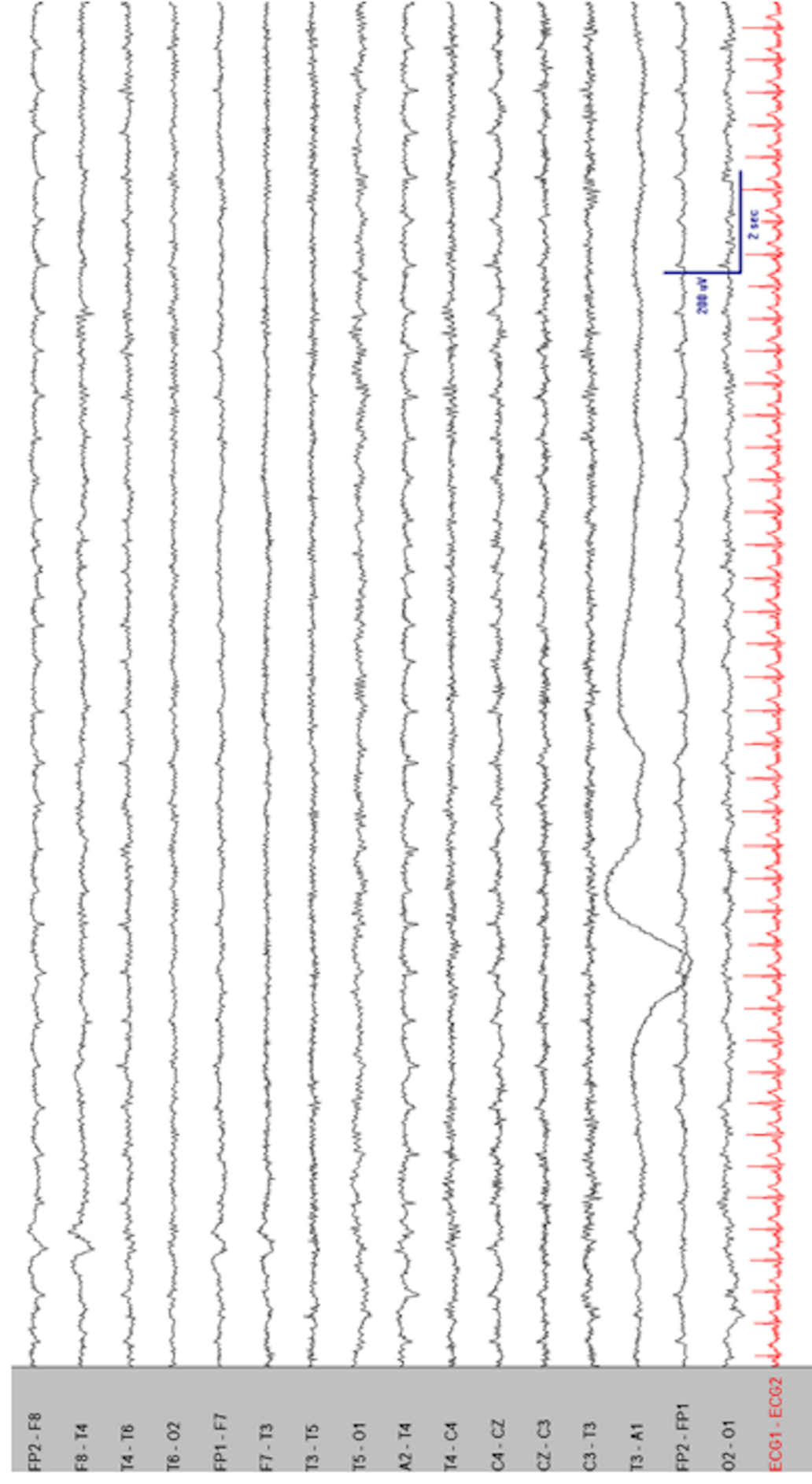

Supplementary Figure 4

Supplement: Supplementary file 4 [file mmc4.pdf]

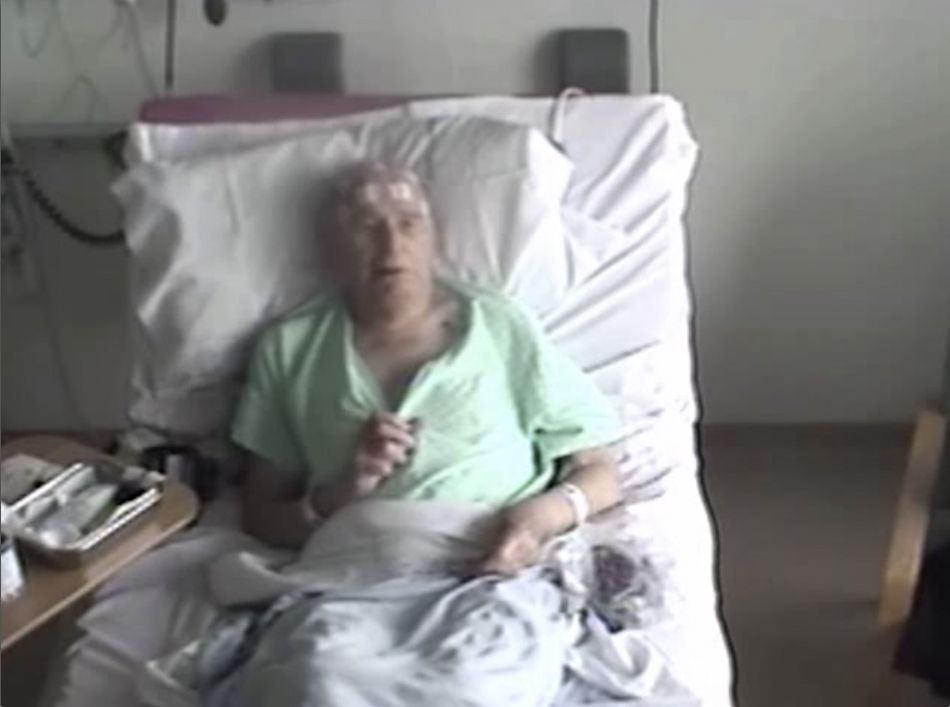

Supplement: Supplementary file 5 [file mmc5.jpg]

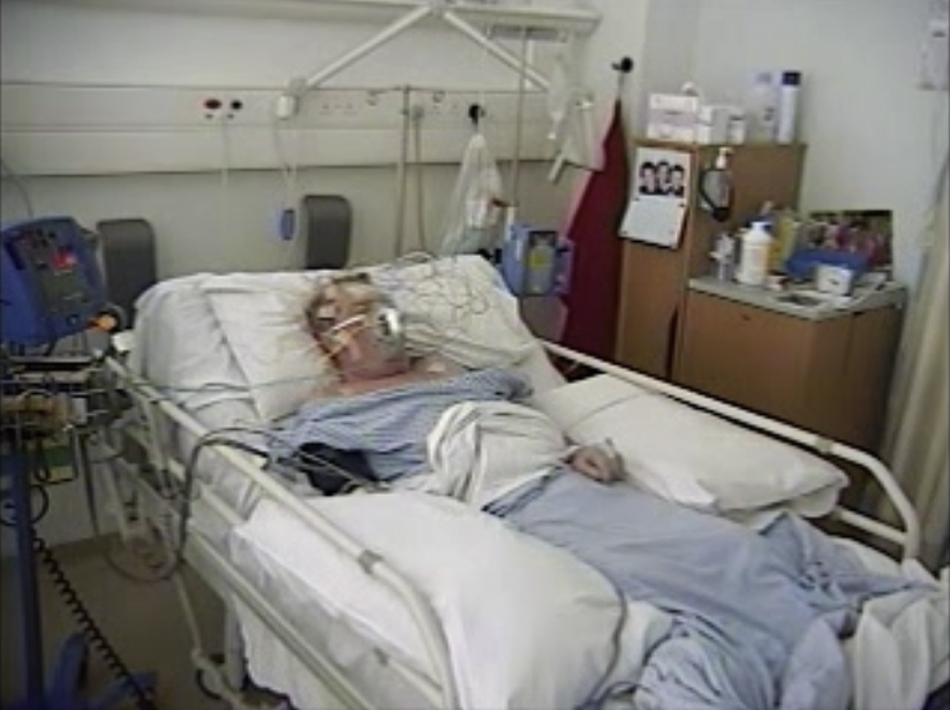

Supplement: Supplementary file 6 [file mmc6.jpg]

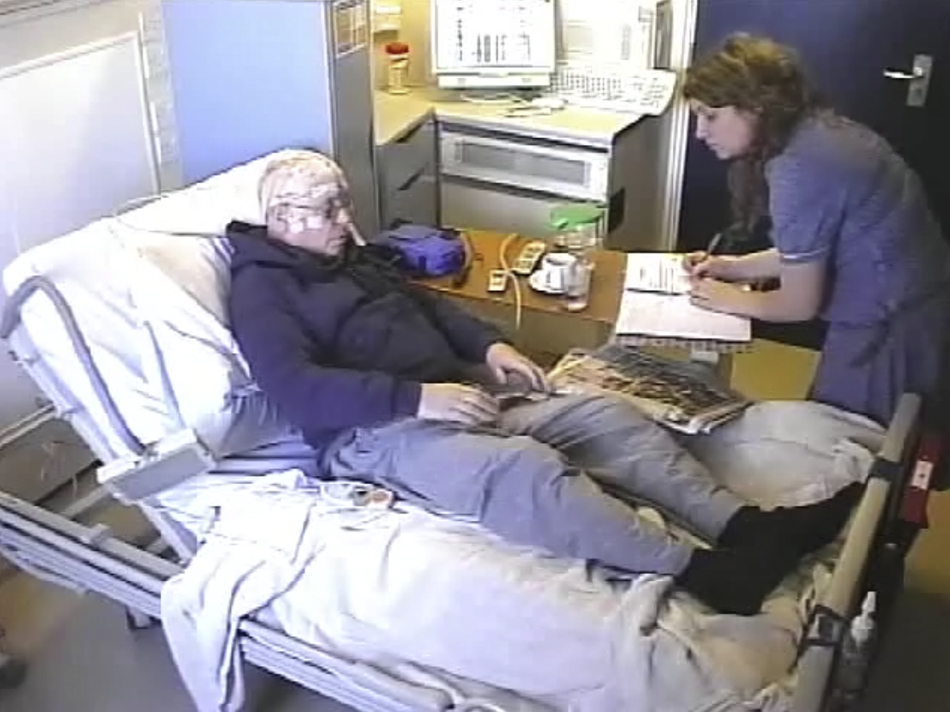

Supplement: Supplementary file 7 [file mmc7.jpg]
